# Exploring the Relationship Between Fragile X Syndrome and Autism: A Bibliometric Analysis of Global Research Trends

**DOI:** 10.7759/cureus.88159

**Published:** 2025-07-17

**Authors:** Alyson Kaplan, Sofia Malik, Nadiya A Persaud, Latha Ganti

**Affiliations:** 1 Research, Ransom Everglades School, Miami, USA; 2 Research, Washington University, St. Louis, USA; 3 Research, Orlando College of Osteopathic Medicine, Winter Garden, USA; 4 Medical Science, Warren Alpert Medical School of Brown University, Providence, USA; 5 Emergency Medicine and Neurology, University of Central Florida, Orlando, USA

**Keywords:** autism spectrum disorder (asd), bibliometric analysis, fragile x messenger ribonucleoprotein-1 (fmr1), fragile x syndrome (fxs), neurodevelopmental disorders

## Abstract

Fragile X syndrome (FXS) is classified as a genetic disorder located in the fragile X messenger ribonucleoprotein-1 (FMR1) gene on the X chromosome. FXS is considered the most prevalent single-gene cause of intellectual disability and autism spectrum disorder (ASD). Understanding the complexity of FXS and ASD requires exploring the correlation between genetics, neuroscience, and behavioral science. This bibliometric analysis explores the data from 3,398 articles collected from the Web of Science database, focusing on FXS and ASD while relating it to the country, institution, keywords, and published data for each article. These publications were imported into VOSviewer to analyze authorship patterns, associated organizations, involved countries, and keywords. The Web of Science database provided graphical figures illustrating the number of publications over the past 25 years and the most prominent funding agencies. Treatments for ASD and FXS often overlap due to their shared characteristics and connections; however, despite numerous clinical trials, no effective treatments have been identified for either condition to date. Although multiple drugs showed potential in preclinical trials, they failed to improve symptoms during the later stages of the trials. This study aims to identify key trends, gaps, and networks with regard to current FXS and ASD research, providing insights to inform future research and the development of effective treatment modalities.

## Introduction and background

Fragile X syndrome (FXS) is a genetic disorder characterized by trinucleotide repeat expansions of the CGG sequence resulting in a mutation in the fragile X messenger ribonucleoprotein-1 (FMR1) located on the X chromosome [1]. The standard number of repeats in the FMR1 gene can range from 5 to 44, but a repetition of 45-54 is considered a grey zone [2]. Furthermore, a pre-mutation is classified as having between 55 and 200 repeats of this trinucleotide, often associated with premature ovarian failure and fragile X-associated tremor/ataxia syndrome (FXTAS), a neurodegenerative disorder caused by damage to the cerebellum and the brain's white matter, leading to associated movement impairments [3]. Those with 200 or more repeats are diagnosed as having a full mutation of FXS [2]. Females with a full mutation are most likely to have milder symptoms and be less affected by this disorder due to presence of an additional nonmutated X chromosome [4]. This mutation results in either a deficiency or absence of the fragile X mental retardation I protein (FMRP) due to the inactivation of a triplet nucleotide repeat expansion, resulting in FXS [5]. Individuals with FXS present with physical abnormalities, such as large ears, large jaw, long face, intellectual disability, anxiety, hyperactivity, and social challenges [1]. FXS affects approximately one in 4,000 to 5,000 individuals worldwide [4]. 

Autism spectrum disorder (ASD), a neurodevelopmental disorder, is characterized by challenges in social interaction and communication, as well as restricted and repetitive behaviors to varying degrees [6]. Those with mild symptoms of ASD may have the ability to live a normal, independent life; however, individuals who are strongly affected may require greater support economically, socially, and educationally, largely impacting their families and society [4]. ASD affects about 1% of the worldwide population [7]. Due to ASD having a broad range of individuals with varying abilities, the prevalence of the diagnosis has increased [8].

The most prevalent single-gene cause of intellectual disability and ASD is FXS [9]. ASD enhances the symptoms of FXS by adding to the severity of the pre-existing symptoms [10]. Those affected by both neurodevelopmental disorders have increased impairment in their communication skills and social interactions, along with more aggressive and repetitive behaviors [11].

A bibliometric analysis is a systematic method that evaluates scientific literature to identify patterns, trends, and key contributions in a specific field [12]. It analyzes bibliographic data from databases such as Web of Science or SCOPUS to examine the connections and impact of published articles. This study aims to provide a detailed understanding of research and its evolution, while highlighting specific areas in the field [13]. Bibliometric analysis enables researchers to understand the field, identify gaps, generate ideas, and position contributions [14].

The interrelationship between genetics, neuroscience, and behavioral science plays a key role in understanding FXS and ASD. This analysis examines the evolution of research on FXS and ASD, highlighting key contributors and institutions that have significantly shaped the field. It also explores prevalent themes and highlights potential research gaps that require attention. 

## Review

Methods

Objectives

This study aimed to examine the volume and trends of publications related to FXS and ASD from 1982 to 2025. Specifically, the analysis focused on authorship patterns, institutional and country-level contributions, citation data, keyword co-occurrence, and research funding sources.

Eligibility Criteria

All articles indexed in the Web of Science Core Collection that included the terms “fragile X” and “autism” in any searchable field (title, abstract, keywords) were eligible for inclusion. Publications of all types (e.g., original articles, reviews, editorials) and all study designs were included. Only English-language documents were analyzed.

Information Sources

The primary data source for this bibliometric analysis was the Web of Science Core Collection.

Search Strategy

A keyword-based search was conducted using the terms “fragile X” and “autism”, yielding a total of 3,398 articles. The search encompassed all publications indexed in Web of Science from 1982 through 2025. The search was limited to English-language results and did not restrict by document type or study design.

Selection Process

All records retrieved by the search strategy were exported from Web of Science for bibliometric analysis. No manual screening or exclusion of articles was conducted after export, as the goal was to assess the global bibliometric landscape of FXS and ASD research in its entirety.

Data Synthesis

The exported publication data were imported into VOSviewer (Leiden University, the Netherlands), a software tool used to construct and visualize bibliometric networks. Graphs were generated to analyze authorship patterns, institutional and organizational affiliations, country-level research output, and keyword co-occurrence networks. A threshold of at least five occurrences was set for inclusion in the visualizations, allowing for the identification of the most prominent contributors in each category. In addition to the visualizations generated using VOSviewer, summary figures from the Web of Science platform were included to illustrate annual publication trends over the past 25 years and identify the top funding agencies supporting research on FXS and ASD.

Results

Author Collaboration

**Figure 1:**
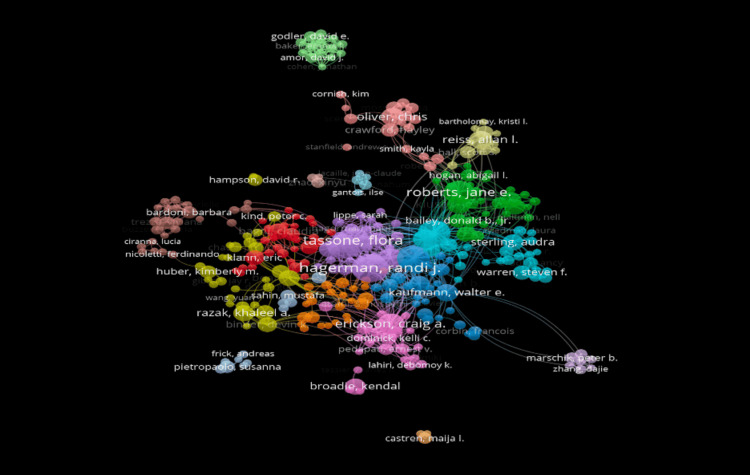
Network visualization of authors’ published documents relating to fragile X syndrome (FXS) and autism spectrum disorder (ASD).

When analyzing author collaboration, each color in the visualization represents a distinct collaborative group, with densely packed nodes indicating stronger interconnectivity. The top three contributing authors in this field are Randi J. Hagerman (87 publications), Flora Tassone (75 publications), and Leonard Abbeduto (70 publications). Randi J. Hagerman emerges as the most prolific author overall (Figure 1).

Institutional Contributions

**Figure 2:**
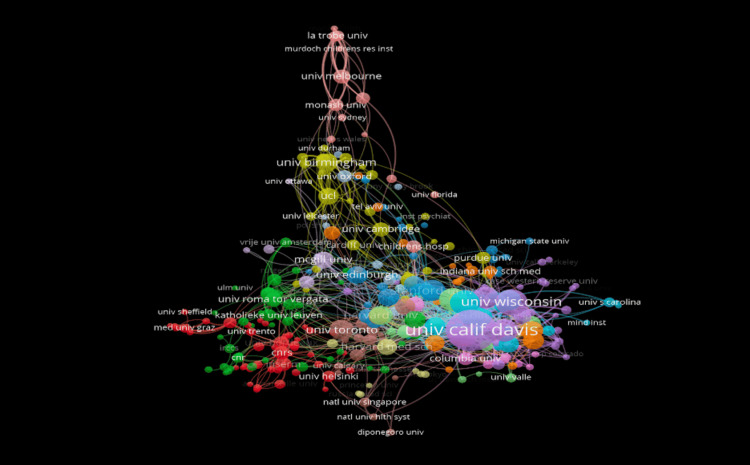
Network visualization of the most prominent organization in the fragile X syndrome (FXS) and autism spectrum disorder (ASD) field.

The University of California, Davis (UCD) is the leading institution with 297 documents and a total link strength of 501. This is followed by the University of Wisconsin (119 documents, 119 links) and the University of North Carolina (102 documents, 176 links). UCD and Wisconsin are clustered together (Purple), while UNC belongs to a separate cluster (Blue). Additional leading U.S. institutions include Johns Hopkins University (90), Rush University (86), Emory University (82), Vanderbilt University (75), Stanford University (74), University of South Carolina (72), and University of Colorado (71) [Figure 2].

Country-Level Output

**Figure 3:**
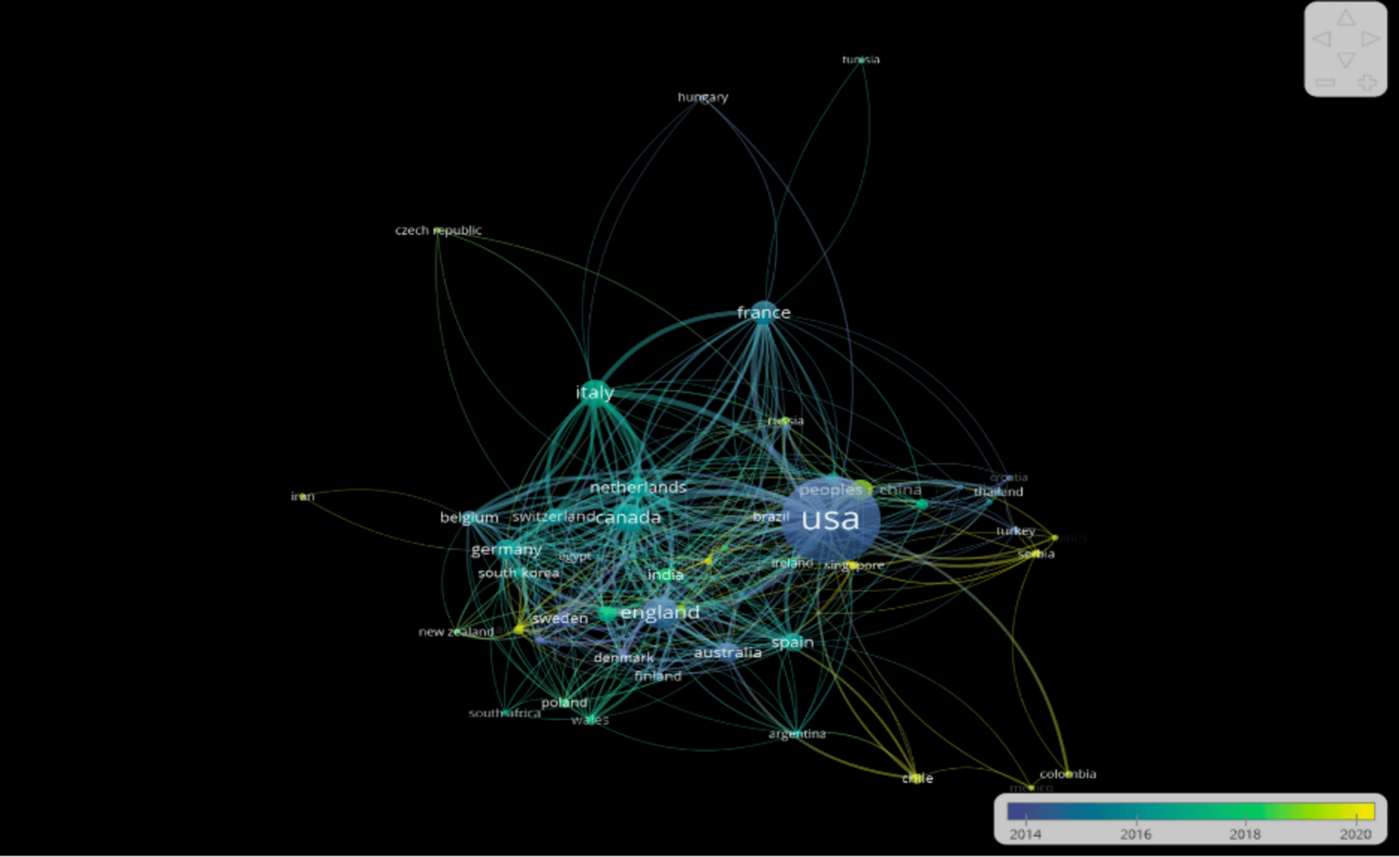
Network visualization of the countries’ number of publications and links in an average timeframe.

The top 10 contributing countries are the USA (2,055 documents), England (278), Canada (235), Italy (214), France (161), China (122), Australia (117), the Netherlands (112), Germany (105), and Spain (93). The United States leads with the highest publication output; however, the average publication year is around 2014, indicating a large portion of earlier work (Figure 3).

Keyword Analysis

**Figure 4:**
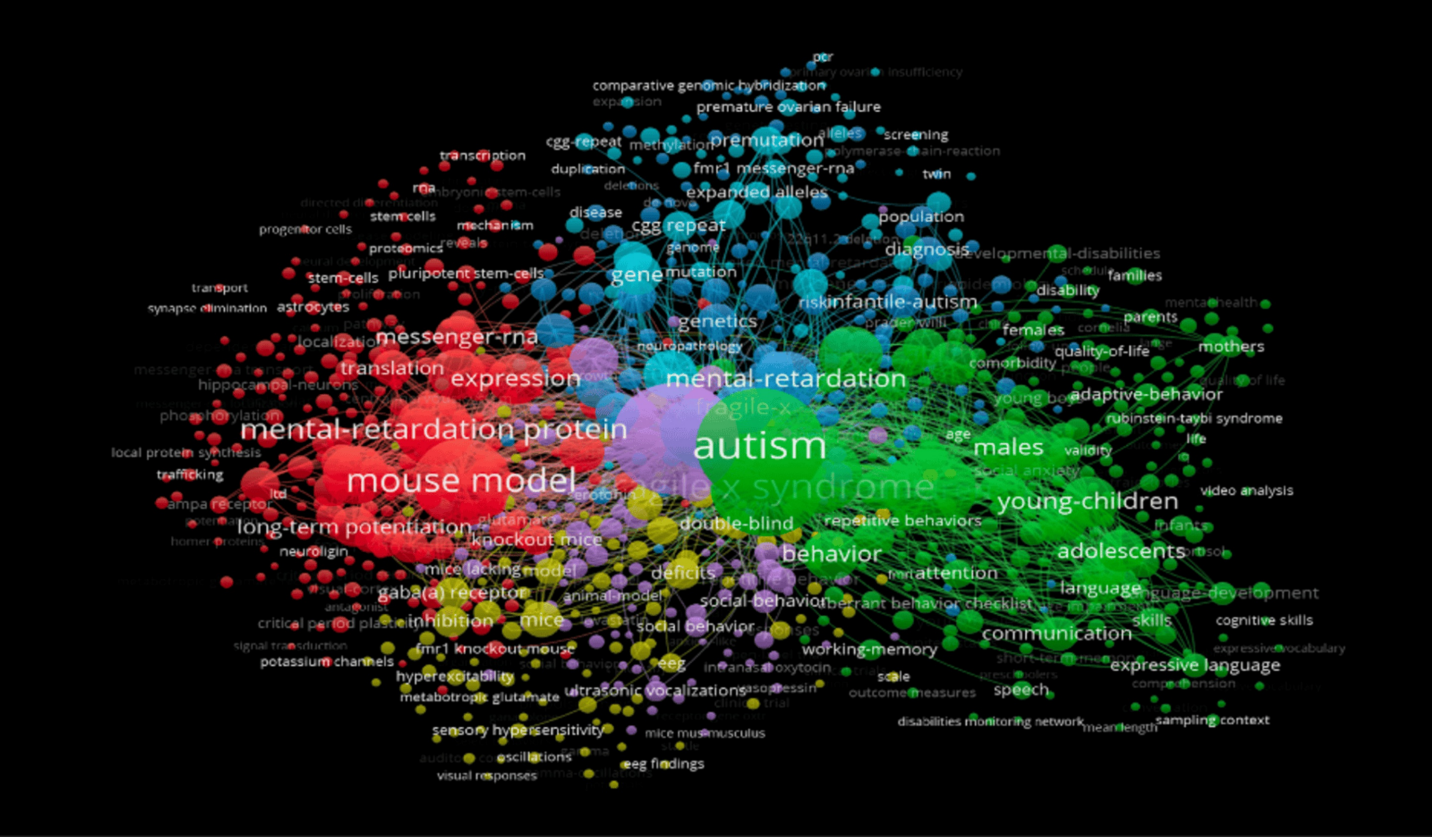
Network visualization of the most prominent keywords relating to fragile X syndrome (FXS) and autism spectrum disorder (ASD).

Keyword co-occurrence mapping reveals the 1,000 most frequently used terms in the dataset. The top 10 keywords are “autism” (1,218), “fragile-x-syndrome” (1,195), “fragile X syndrome” (945), “mouse model” (727), “children” (626), “autism spectrum disorder” (577), “mental-retardation protein” (456), “autism spectrum disorders” (380), “synaptic plasticity” (328), and “mental-retardation” (306) (Figure 4).

Publication Trends

**Figure 5:**
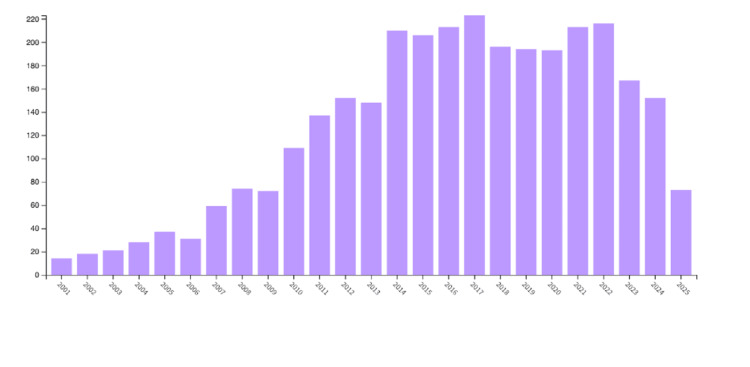
Network visualization of the number of publications per year from 2001 to 2025.

Figure 5 shows the annual publication volume from 2001 to 2025. The year 2017 had the highest output, comprising 6.561% of total publications. There was a steady increase in publications from 2001 to 2022, peaking in 2017, followed by a decline from 2023 to present.

Funding Sources

**Figure 6:**
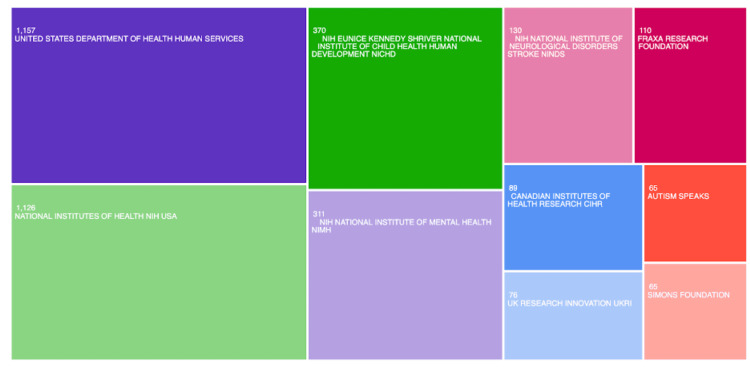
Network visualization of the most influential funding agencies for fragile X syndrome (FXS) and autism spectrum disorder (ASD) research.

The United States Department of Health Human Services (N = 1,157), National Institutes of Health NIH USA (N = 1,126), NIH Eunice Kennedy Shriver National Institute of Child Health Human Development NICHD (N = 370), NIH National Institute of Mental Health NIMH (N = 311), and NIH National Institute of Neurological Disorders Stroke NINDS (N = 130) represent the leading funding agencies for research on FXS and ASD. All of the top five agencies are located in the United States, providing strong evidence to support the United States’ strong influence on FSX and ASD research (Figure 6).

Discussion

Key Findings

This study analyzed 3,398 publications exported from the Web of Science database. As shown in Figures 1-3, Randi J. Hagerman of the UCD was shown to be the most prominent published author in FXS- and ASD-related research. The majority of the analyzed publications were found to be from UCD, portraying a strong correlation between the organization and Hagerman’s research (Figure 2). The most dominant keywords were found to be “autism,” “fragile-x-syndrome,” “fragile x syndrome,” “mouse model,” and “children” (Figure 4). Hagerman’s publications most often include the keywords “fragile X” and “autism,” influencing the bibliometric data due to the large quantity of her publications [15,16]. In addition to her many citations, Hagerman also has many collaborations with authors, including Flora Tassone, the second most dominant author in the field, and Paul Hagerman, Randi Hagerman’s husband (Figure 1) [17,18]. UCD is the leading organization in FXS and ASD research, with the highest number of publications, allowing the United States to pave the way for innovation and discovery. As a result, the United States allocates the greatest number of resources dedicated to understanding FXS and ASD, further emphasizing its role in advancement and impact (Figures 3, 6). The United States Department of Health and Human Services (USDHHS) was shown to be the leading funding agency, followed by the NIH as the second regarding FXS and ASD research (Figure 6). Publication trends remained consistently stable from 2014 to 2022, with a stark decrease from 2022 onward (Figure 5). This directly correlates with significant institutional funding cuts, leading to insufficient research funding and decreased publications [19]. 

Current treatments for ASD coincide with the treatments for FXS due to their strong relationship and similarities with one another. Various treatments include serotonergic medications (e.g., sertraline), atypical antipsychotics (e.g., risperidone and aripiprazole), stimulant medications (e.g., amphetamines and methylphenidate derivatives), alpha-2-adrenergic agonists (e.g., guanfacine and clonidine), melatonin, N-acetylcysteine, dietary supplements, oxytocin, bumetanide, metformin, lovastatin, cannabidiol, arbaclofen, trofinetide, phosphodiesterase 4D inhibitors, anavex 2-73, and gene therapy. These medications assist with social functioning, cognitive function, motor function, visual function, sleeping, lowering cholesterol levels, improving synaptic connections, oxidative stress, and anxiety [20,21]. 

In recent years, there have been numerous clinical trials proposing a solution to FXS and ASD; however, none have yet to truly deliver an evidence-based, effective treatment. Although some drugs, particularly aripiprazole and risperidone, reduce the symptoms of autism, they fail to resolve the core symptoms of ASD. In addition to no solution being discovered for ASD, no effective treatment has succeeded for FXS. Various drugs have shown promising potential in the preclinical trials, but did not improve or treat the symptoms of FXS and ASD in the later stages of the research (e.g., donepezil, memantine, metformin, and oxytocin) [21]. Due to the lack of resources, organizations and institutions need more funds to facilitate more research on treatments for FXS and ASD.

Strengths and Limitations

The generated graphs may not accurately represent the total number of publications per author because some authors publish under multiple different names. For each term, there may be another name associated with it or structured differently (e.g., fragile x syndrome and fragile-x-syndrome, autism and autism spectrum disorder). Due to the USA being the most prominent country in this field, the data from available studies may not represent the global population with FXS. This research is only as accurate as the diagnosed individuals with FXS. This bibliometric analysis only used data from the Web of Science database, limiting the data collected. The name for the FMR1 gene is currently known as “fragile X messenger ribonucleoprotein-1” gene, replacing the old name “fragile X mental retardation-1” in 2022.

## Conclusions

This bibliometric analysis investigated 3,398 articles retrieved from the Web of Science database, with a focus on FXS and ASD. This study mapped key research contributors by analyzing leading countries (e.g., USA), institutions (e.g., UCD), frequently used keywords (e.g., autism, fragile-x-syndrome), and publication trends. The findings emphasize the critical need for continued research to advance the development of effective treatments and long-term solutions for both FXS and ASD.
